# Bacterial cell division is recognized by the septin cytoskeleton for restriction by autophagy

**DOI:** 10.1080/15548627.2019.1586499

**Published:** 2019-03-11

**Authors:** Sina Krokowski, Serge Mostowy

**Affiliations:** Department of Immunology & Infection, London School of Hygiene & Tropical Medicine, London, UK

**Keywords:** Autophagy, cardiolipin, cytoskeleton, membrane curvature, mitochondria, septins, *Shigella*

## Abstract

Septins are cytoskeletal proteins widely recognized for their role in eukaryotic cell division. Septins also assemble into cage-like structures that entrap cytosolic *Shigella flexneri* targeted to macroautophagy/autophagy. Although the *Shigella* septin cage was discovered ~10 y ago, how septins recognize *Shigella* was poorly understood. We found that septins are recruited to regions of micrometer-scale curvature presented by dividing bacterial cells, and cardiolipin (a curvature-specific phospholipid) promotes septin recruitment to these regions. Chemical manipulation of bacteria revealed that following recruitment, septins assemble into cages around growing bacterial cells. Once assembled, septin cages inhibit *Shigella* cell division by autophagy and fusion with lysosomes. Thus, recognition of dividing bacterial cells by the septin cytoskeleton targets intracellular pathogens to antibacterial autophagy.

*Shigella flexneri* is a Gram-negative enteroinvasive bacterial pathogen used as a paradigm to study autophagic elimination of bacteria (xenophagy). Following host cell invasion, *Shigella* escape from the phagosome to proliferate in the cytosol and polymerize actin tails for dissemination. To defend against *Shigella* proliferation, host cells use autophagy receptors (including SQSTM1/p62) to recognize ubiquitinated substrates or damaged cellular membrane surrounding bacteria, and form autophagosomes. To prevent bacterial dissemination, host cells use the septin cytoskeleton to entrap actin-polymerizing bacteria in cage-like structures. Septins (SEPT1-SEPT14 in humans) are GTP-binding proteins in which subunits form hetero-oligomeric complexes that assemble into non-polar filaments and rings. The *Shigella*-septin cage was first described ~10 y ago, yet we still lack fundamental insights into how septins recognize bacteria for cage entrapment. We recently discovered that septin recognition of micrometer-scale curvature and growth during bacterial cell division is used by host cells to target *Shigella* to antibacterial autophagy [1].

Septins recognize areas of the plasma membrane presenting micrometer-scale curvature, including the cytokinetic furrow and the base of cell protrusions (e.g., cilia, dendritic spines). Considering this, we hypothesized that septins may be recruited to the membrane of *Shigella* cells (~1 μm in diameter) in a curvature-dependent manner. To test this, we examined the recruitment of GFP-SEPT6 to *Shigella*-mCherry using time-lapse microscopy. For the majority of entrapped bacteria, we observed that GFP-SEPT6 is recruited to the bacterial cell division site and/or poles (i.e., regions of high curvature) before assembling into cage-like structures. Bacterial invagination at the division site is driven by FtsZ, the bacterial homolog for tubulin which forms the cytokinetic Z-ring. To follow the division site of intracellular bacteria during infection, we expressed an inducible FtsZ-GFP fusion in *Shigella* and used a variety of time-lapse and high-resolution microscopy techniques. From this, we found that RFP-SEPT6 and SEPT7 alignment to FtsZ-GFP is highly conserved, and conclude that bacterial curvature generated by Z-ring constriction promotes septin recruitment.

Considering that septins bind anionic phospholipids of eukaryotic membranes, we reasoned that septins might also bind anionic phospholipids of bacterial membranes. In rod-shaped bacteria, cardiolipin is enriched at the bacterial cell division site and poles. We purified recombinant septin proteins and tested for lipid binding in vitro using membrane lipid strips and purified cardiolipin from *Escherichia coli*; these results showed that SEPT2/6/7, SEPT6/7, and SEPT9 can specifically bind cardiolipin. We next performed liposome flotation assays using purified recombinant septin proteins and total lipid extracts from *Shigella* with or without cardiolipin. In this case, SEPT2/6/7 clearly binds vesicles made from bacterial total lipid extracts, and binds significantly more to vesicles produced from wild-type *Shigella* than *Shigella* lacking cardiolipin (cardiolipin KO) . Consistent with this, quantitative microscopy revealed that cardiolipin KO *Shigella* are significantly less entrapped in SEPT7 cages than wild-type *Shigella*. To dissect the role of curvature and cardiolipin in septin recruitment, we prepared supported bacterial lipid bilayers on beads of different diameter and compared SEPT2/6/7 recruitment to beads coated with *S. flexneri* lipids with or without cardiolipin. Here, we found that SEPT2/6/7 preferentially associate with 1-μm beads, and cardiolipin promotes the recruitment of SEPT2/6/7 to bacterial curvature.

What are the bacterial determinants required for cage assembly to proceed after septin recruitment? To address this, we used antibiotics against intracellular bacteria targeting different processes of bacterial cell division. Under conditions in which bacterial cell growth is inhibited, SEPT7 cages are rarely observed. In this case, time-lapse microscopy showed that GFP-SEPT6 is transiently recruited to poles of non-growing bacteria but fails to assemble into cages. These data suggested that bacterial curvature is required for septin recruitment, and bacterial growth is required for septin cage assembly. To test this, we treated infected cells with cephalexin, an antibiotic that prevents bacterial cell separation and promotes filamentation. Here, the percentage of *Shigella* entrapped in SEPT7 cage-like structures is significantly increased, and time-lapse microscopy revealed that GFP-SEPT6 recruitment happens to bacterial curvature prolonged by cephalexin before it inhibits cell separation. Collectively, these results show that following recruitment, septins assemble into cages around growing bacterial cells.

Previous work suggested that ~50% of SEPT7-cage entrapped bacteria are metabolically inactive. We thus asked whether septin cages can inhibit the division of entrapped *Shigella*, and observed that the vast majority of RFP-SEPT6 cage-entrapped *Shigella* lose their Z-ring and fail to divide. To test the role of autophagy in this process, we quantified Z-ring positive *Shigella* recruiting septins (SEPT7 and RFP-SEPT6) and/or autophagy markers SQSTM1 and LC3B. We found that *Shigella* recruiting both SEPT7 and SQSTM1 are less Z-ring positive, as compared with bacteria recruiting SEPT7 but not SQSTM1. Moreover, we found that *Shigella* recruiting both SEPT7 and SQSTM1 are less Z-ring positive, as compared with bacteria recruiting SQSTM1 but not SEPT7. Similar results are obtained using RFP-SEPT6 and LC3B. These results show that septins are necessary but not sufficient for *Shigella* Z-ring loss, and that septins and autophagy act interdependently to restrict bacterial cell division. To test whether this restriction is dependent on fusion with lysosomes, we treated infected cells with chloroquine to inhibit lysosome fusion. In this case, SEPT7-cage entrapped *Shigella* fail to lose their Z-ring. Collectively, these results demonstrate that septin cages entrap actively dividing bacterial cells and use autophagy to prevent further division events by fusion with lysosomes (Figure 1).

**Figure 1.**
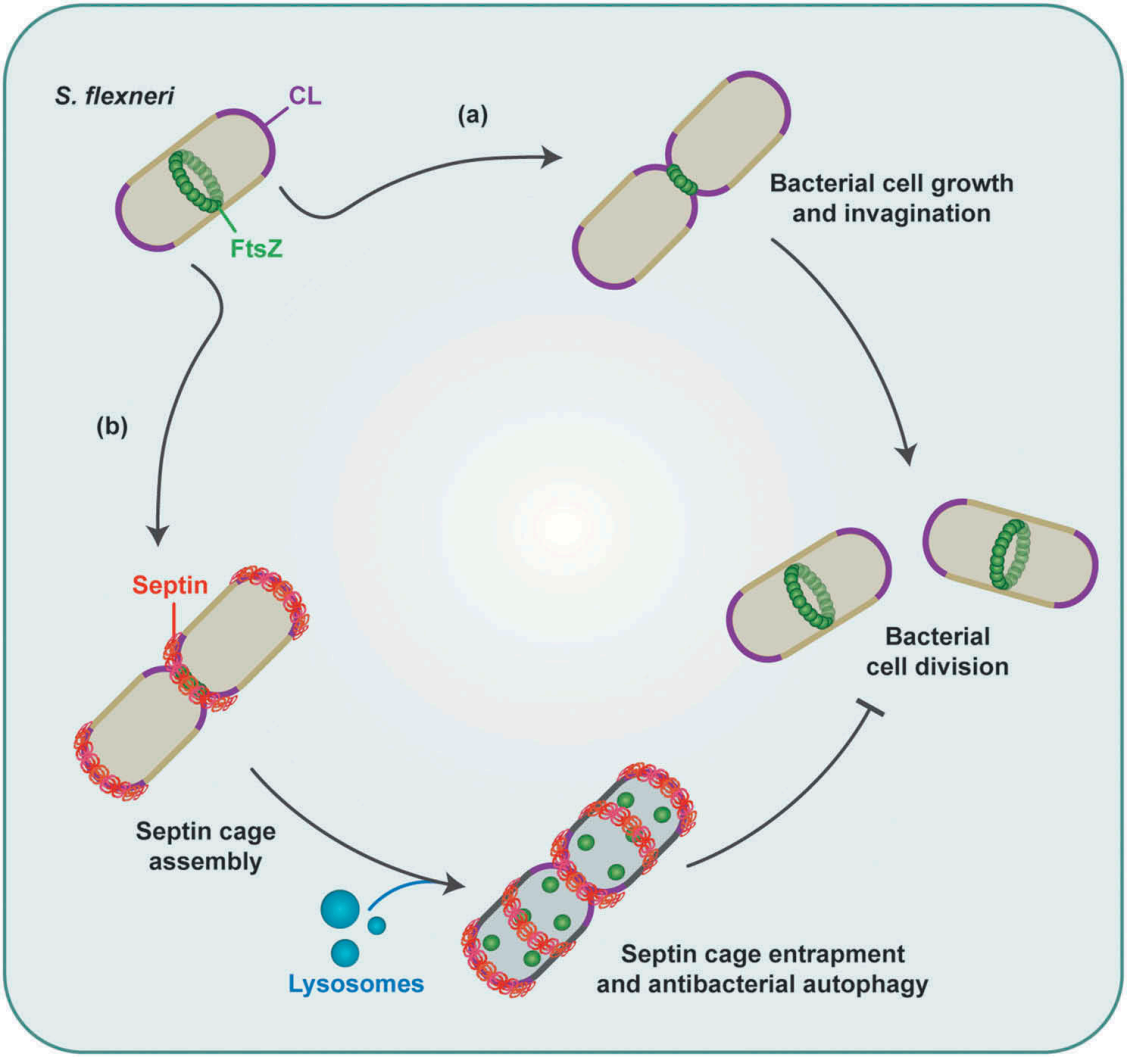
Bacterial factors required for septin cage entrapment. In actively dividing *Shigella*, FtsZ assembles into the cytokinetic Z-ring at the bacterial midcell. (a) In the cytosol of infected host cells, *Shigella* cells grow, and constriction of the Z-ring leads to membrane invagination, and finally bacterial cell division. (b) Septins recognize regions of high curvature at the bacterial cell division site and/or poles, where they bind cardiolipin (CL). Septin cages assemble around growing bacterial cells, and inhibit bacterial cell division via autophagy and fusion with lysosomes.

We have uncovered a mechanism used by host cells to recognize bacterial cell division for restriction by autophagy. In addition to *S. flexneri*, we observed septin recruitment to micrometer-scale curvature presented by a variety of important human pathogens including *Shigella sonnei, Pseudomonas aeruginosa* and *Staphylococcus aureus*. Considering that mitochondria are viewed as ancient bacteria, future investigation of mitochondria-septin interactions in vitro and in vivo may discover unexpected roles for micrometer-scale curvature and cardiolipin underlying mitochondrial dynamics and mitophagy. Having identified a new link between cell division and cell-autonomous immunity, we conclude that bacterial curvature and growth are fundamental danger signals used by host cells to recognize invasive bacterial pathogens for restriction by autophagy. It will thus be of great interest to determine how this natural antibacterial system can be used to stop infection.
